# Immunogenicity of Infliximab Among Patients With Behçet Syndrome: A Controlled Study

**DOI:** 10.3389/fimmu.2020.618973

**Published:** 2020-12-22

**Authors:** Sinem Nihal Esatoglu, Fatma Nihan Akkoc-Mustafayev, Yesim Ozguler, Fatma Ozbakır, Okan K. Nohut, Dilsen Cevirgen, Vedat Hamuryudan, Ibrahim Hatemi, Aykut Ferhat Celik, Hasan Yazici, Gulen Hatemi

**Affiliations:** ^1^ Division of Rheumatology, Department of Internal Medicine, Cerrahpasa Medical School, Istanbul University-Cerrahpasa, Istanbul, Turkey; ^2^ Department of Internal Medicine, Cerrahpasa Medical School, Istanbul University-Cerrahpasa, Istanbul, Turkey; ^3^ Central Research Laboratory, Cerrahpasa Medical School, Istanbul University-Cerrahpasa, Istanbul, Turkey; ^4^ Division of Gastroenterology, Department of Internal Medicine, Cerrahpasa Medical School, Istanbul University-Cerrahpasa, Istanbul, Turkey

**Keywords:** infliximab · Remicade, immunogenicity, Behçet disease, drug antibodies, drug level testing

## Abstract

**Background:**

Immunogenicity of tumor necrosis factor alpha inhibitors (TNFis) has been recognized as an important problem that may cause loss of efficacy and adverse events such as infusion reactions. TNFis are being increasingly used among patients with Behçet syndrome (BS) and scarce data exist on this topic.

**Objective:**

We aimed to investigate the prevalence of anti-infliximab (IFX) antibodies in patients with Behçet syndrome together with suitable controls.

**Methods:**

We collected serum samples from 66 consecutive Behçet syndrome patients (51 M, 15 F, mean age 37 ± 9 years) who were treated with IFX. Additionally, similarly treated 27 rheumatoid arthritis, 53 ankylosing spondylitis, 25 Crohn’s disease patients, and 31 healthy subjects were included as controls. Samples were collected just before an infusion, stored at −80°C until analysis, and serum IFX trough levels and anti-IFX antibodies were measured by ELISA. We used a cut-off value of 1 μg/ml for serum IFX trough level, extrapolating from rheumatoid arthritis studies.

**Results:**

Anti-IFX antibodies were detected in four (6%) Behçet syndrome, five (18.5%) rheumatoid arthritis, three (12%) Crohn’s disease, and one (2%) ankylosing spondylitis patient. The median serum IFX trough level was significantly lower in patients with anti-IFX antibodies compared to those without antibodies [2.32 (IQR: 0.6–3.6) vs. 3.35 (IQR: 1.63–5.6); p = 0.019]. The serum IFX trough level was lower than the cut-off value in 6/13 (46%) patients with anti-IFX antibodies and in 25/158 (16%) patients without anti-IFX antibodies (p = 0.015). Among the four Behçet syndrome patients with anti-IFX antibodies, two experienced relapses and two had infusion reactions.

**Conclusions:**

Immunogenicity does not seem to be a frequent problem in Behçet syndrome patients treated with IFX, but may be associated with relapses and infusion reactions, when present.

## Introduction

Tumor necrosis factor (TNF)-alpha, a pro-inflammatory cytokine, has a crucial role in the pathogenesis of chronic inflammatory and immune-mediated diseases such as rheumatoid arthritis, ankylosing spondylitis, Crohn’s disease, ulcerative colitis, psoriasis and psoriatic arthritis (1). Tumor necrosis factor alpha inhibitors (TNFi) have been the first biologic agents targeting a specific inflammatory mediator for the treatment of inflammatory rheumatic diseases and they provided a breakthrough in the management of these diseases (2). However, they may generate an immune response leading to the development of anti-drug antibodies (ADAs). The formation of immune complexes consisting of ADAs and drugs may influence pharmacokinetics, bioavailability and efficacy of TNFi (3). This process is called immunogenicity and is considered to be responsible for secondary failure of TNFi as well as therapy-limiting adverse events such as infusion reactions, paradoxical inflammation and triggering of autoimmune diseases (4).

Immunogenicity has mainly been a concern with monoclonal anti-TNF antibodies especially with infliximab (IFX) (5, 6). Immunogenicity of IFX has been studied in nearly 500 studies, in different patient populations including patients with rheumatoid arthritis, ankylosing spondylitis, juvenile idiopathic arthritis, psoriasis, psoriatic arthritis, ulcerative colitis, and Crohn’s disease (5). The prevalence of ADAs against IFX shows wide variation from 3% to 80%, varying across the studies and studied diseases (5). The high frequency of ADAs against IFX compared to that of other TNFi is mostly due to its 25% murine sequences which may lead to the production of human anti-IFX antibodies (6). Development of ADAs has been found to be highly associated with both loss of efficacy and adverse events. In a systematic literature review of 443 studies, ADA-positive patients were reported to have significantly lower serum drug concentrations compared to ADA-negative patients and such patients were prone to inadequate response and adverse drug reactions (5). These findings led the Food and Drug Administration (7) and the European Medicines Agency (8) to include immunogenicity testing as a mandatory part of drug safety assessment. Moreover, monitoring of ADAs and biologic concentrations are recommended to guide treatment changes in inflammatory bowel disease guidelines (9). Although therapeutic drug monitoring is not currently suggested in the published guidelines on rheumatic diseases, many reviews recommend its use, especially in rheumatoid arthritis (10).

IFX has been widely used off-label for the management of Behçet syndrome (11). In the 2018 update of the European League Against Rheumatism (EULAR) recommendations, monoclonal anti-TNF antibodies were recommended for all severe and refractory manifestations of Behçet syndrome including mucocutaneous, eye, vascular, gastrointestinal, and central nervous system involvement (12). There have been two previous reports on immunogenicity of TNFi in patients with Behçet syndrome. The first was a case series of nine patients treated with adalimumab. It was reported that none of these patients developed ADA during a mean treatment duration of 5 years (13). The only other report is a congress abstract that was presented 5 years ago, which showed that 18 of the 160 (11.3%) studied patients had anti-IFX antibodies and the presence of antibodies was associated with infusion reactions, low IFX trough levels, and therapeutic failure. Details of this analysis were not available in the abstract, and there were no controls (14).

Considering the potential impact of immunogenicity on the efficacy and safety of TNFi, wide variation in the prevalence of ADAs across inflammatory diseases, and the paucity of data regarding immunogenicity in Behçet syndrome, we aimed to investigate the prevalence of ADAs against IFX in patients with Behçet syndrome together with suitable controls.

## Materials and Methods

### Patient Population

Between June 2016 and January 2017, we collected serum samples from 66 consecutive Behçet syndrome patients (41 with eye involvement, 11 with vascular involvement, 8 with central nervous system involvement, 2 with both mucocutaneous and joint involvement, 2 with venous ulcer, and 2 with both eye and vascular involvement) receiving originator IFX (Remicade^®^), together with 27 rheumatoid arthritis, 53 ankylosing spondylitis, and 25 Crohn’s disease patients who received originator IFX (Remicade^®^) in our rheumatology infusion unit during the same time. Additionally, we included 31 healthy controls in order to test the reliability of the kits for false positive results. Patients who had received at least four infusions of IFX were included. Patients with Behçet syndrome fulfilled the International Study Group Criteria for Behçet’s Disease (15), patients with rheumatoid arthritis fulfilled the 2010 American College of Rheumatology/EULAR criteria (16), patients with ankylosing spondylitis fulfilled the modified New York criteria (17), and patients with Crohn’s disease were diagnosed by two expert gastroenterologists (AFC and IH).

### IFX Dose and Regimen

IFX was given with a dose of 5 mg/kg as an induction regimen at week 0, 2, and 6 followed by maintenance treatment every 6–8 weeks for patients with Behçet syndrome, ankylosing spondylitis and Crohn’s disease. IFX dose was 3 mg/kg in patients with rheumatoid arthritis.

### Data Collection

Demographic and clinical characteristics, concomitant immunosuppressive or disease-modifying antirheumatic drugs (DMARDs), the number of IFX infusions, and previous infusion reactions to IFX were obtained from patients’ charts. Disease activity was assessed using the Behçet’s Syndrome Activity Scale (BSAS) for Behçet syndrome patients, the Bath Ankylosing Spondylitis Disease Activity Index (BASDAI) for ankylosing spondylitis patients, the Disease Activity Score-28 (DAS-28) for rheumatoid arthritis patients and Clinical Disease Activity Index (CDAI) for Crohn’s disease patients at the time of serum collection. BSAS ranges between 0 and 100 with higher scores indicating more active disease. A DAS-28 score of greater than 5.1 indicates active disease, less than 3.2 indicates low disease activity, and less than 2.6 indicates remission. BASDAI values of greater than 4 indicate active disease. CDAI score ranges from 0 to 600. CDAI scores between 150 and 219 indicate mildly active disease, scores between 220 and 450 indicate moderately active disease and scores over 450 indicate severely active disease.

After serum sampling, we continued to follow Behçet syndrome patients for treatment efficacy and infusion reactions until October 2019. Infusion reactions were defined as events occurring during an infusion which required either cessation of the IFX infusion or the administration of parenteral medication including diphenhydramine and/or methylprednisolone. Loss of efficacy was defined as new disease activity that required increasing doses of IFX, decreasing interval of IFX infusions, adding new DMARDs, increasing glucocorticoid dose and/or switching to another biologic agent for better disease control.

### Measurement of Serum IFX Trough Levels and Anti-IFX Antibodies

Serum samples were collected from all patients just before an infusion. Additionally, in order to test for the consistency of ADAs and IFX through levels at different time points, we collected serum samples before the next infusion from 27 Behçet syndrome patients for ADAs and 14 days after the infusion from 5 Behçet syndrome patients for IFX trough levels who gave informed consent. After centrifugation, all serum aliquots were immediately frozen and stored at −80°C until analysis. Serum IFX trough levels and anti-IFX antibodies were measured by enzyme-linked immunosorbent assay (ELISA) at the same time.

Measurement of serum IFX (Remicade) (Q-INFLIXI) and antibody to IFX (Q-ATI) (SHIKARI ^®^- Matriks Biotek^®^, Turkey) levels were determined by solid phase enzyme linked immunosorbent assays (ELISA) based on the same sandwich principle (18, 19).

During the Q-INFLIXI- or Q-ATI ELISA’s analysis, in the first period, samples (serum), specific standards and controls were incubated, in the microtiter plate coated with the reactant for IFX (Remicade) or antibody to IFX. After incubation (30 and 60 min, respectively), the wells were washed. Peroxidase Conjugated probes were added and bound to IFX or antibody to IFX, that captured by the reactant on the surface of the wells. Following incubation (30 and 60 min, respectively), wells were washed and the bound enzymatic activity was detected by addition of chromogen-substrate. The color developed was proportional to the amount of IFX or antibody to IFX, in the samples, specific standards and controls (10 and 20 min, respectively). Finally, the reaction terminated with an acidic stop solution. Optical density was measured with a photometer at 450/650 nm within 30 min after pipetting of the Stop Solution.

Q-IFX levels of all samples were determined by using the standard curves and concentrations were given in µg/ml. The levels of Q-ATI, according the cutoff control value (3), If “OD 450/650 Sampling/Average OD 450/650 of Negative Controls” was ≥3, the sample was considered positive. If it was <3, the sample was considered negative.

### Ethics

This study was conducted in accordance with the Declaration of Helsinki on Ethical Principles and was approved by the ethics committee of Cerrahpasa Medical School, Istanbul University-Cerrahpasa (83045809/852). The study was funded by Istanbul University, Scientific Research Projects Coordination Unit (Project no: 57420). Informed written consent was obtained from all participants.

### Statistics

Statistical analyses were performed using SPSS 20.0. Descriptive statistics have been used to describe variables. Continuous variables were represented as median and interquartile range (IQR) unless the data had normal distribution. We used a cut-off value of 1 μg/ml for serum IFX trough level, extrapolating from rheumatoid arthritis studies (20). Comparison of continuous variables of patients with a low serum IFX trough level and those with normal levels was done using a Mann-Whitney U test. Chi-square test was used for comparison of categorical variables. A *p* value < 0.05 was considered significant.

## Results

### Patient Characteristics

Demographics, disease characteristics, concomitant use of immunosuppressives and DMARDs, disease activity scores, previous infusion reactions to IFX, and the mean number of IFX infusions at the time of serum sampling are shown in **Table 1**. Previous infusion reactions had occurred in nine (14%) Behçet syndrome, six (22%) rheumatoid arthritis, four (7.5%) ankylosing spondylitis, and five (20%) Crohn’s disease patients. Mean number of IFX infusions before serum sampling was 19 ± 14 in Behçet syndrome patients, 21 ± 13 in rheumatoid arthritis patients, 33 ± 18 in ankylosing spondylitis patients and 19 ± 21 in Crohn’s disease patients. Number of patients who used concomitant DMARDs was 46 (70%) in Behçet syndrome, 17 (63%) in rheumatoid arthritis, 7 (13%) in ankylosing spondylitis, and 22 (88%) in Crohn’s disease.

**Table 1:** Demographic and characteristics of the included subjects.

	Behçet syndrome(n = 66)	Ankylosing spondylitis(n = 53)	Crohn’s disease(n = 25)	Rheumatoid arthritis(n = 27)	Healthy subjects (n = 31)
Mean age (SD)	37 (9)	45 (12)	39 (13.5)	55 (12)	40 (8)
Male gender (n, %)	51 (77)	40 (76)	14 (56)	5 (18.5)	14 (45)
Patients with previous infusion reactions to IFX (n, %)	9 (14)	4 (7.5)	5 (20)	6 (22)	N/A
Patients using glucocorticoid (n, %)	22 (33.3)	3 (6)	2 (8)	9 (33.3)	N/A
Patients using colchicine (n, %)	26 (39)	N/A	N/A	N/A	N/A
Patients with concomitant immunosuppressive or DMARD use* (n, %)	46 (70)	7 (13)	22 (88)	17 (63)	N/A
Azathioprine	42	0	20	0	N/A
Methotrexate	0	2	2	12	N/A
Cyclosporine-A	6	0	0	0	N/A
Leflunomide	0	0	0	6	N/A
Sulfasalazine	0	5	0	4	N/A
Mycophenolate mofetil	3	0	0	0	N/A
Mean (SD) number of IFX infusions	19 (14)	33 (18)	19 (21)	21 (13)	N/A
Mean (SD) disease activity scores	BSAS 3.5 (4.5)	BASDAI 3.2 (2)	CDAI 74 (49)	DAS-28 3.1 (0.8)	N/A

N/A, not applicable; IFX, infliximab; SD, standard deviation; DMARD, disease modifying antirheumatic agent; BSAS, Behçet’s Syndrome Activity Scale; BASDAI, Bath Ankylosing Spondylitis Disease Activity Index; DAS-28, Disease Activity Score-28; CDAI, Clinical Disease Activity Index.

*Percentages may not add up to 100% due to rounding.

The mean BSAS score of Behçet syndrome patients was 4.4 ± 6.4. All patients with Behçet syndrome had BSAS scores over 0 showing that none of them were in complete remission. Among the 27 RA patients, 4 were in remission, 8 had low disease activity and 15 had moderate disease activity according to the DAS-28. Eighteen of the 53 ankylosing spondylitis patients had active disease (BASDAI ≥ 4). Apart from three patients with mildly active disease, all Crohn’s disease patients were in remission according to CDAI scores.

### Anti-IFX Antibodies and Serum Trough IFX Levels

**Figure 1:**
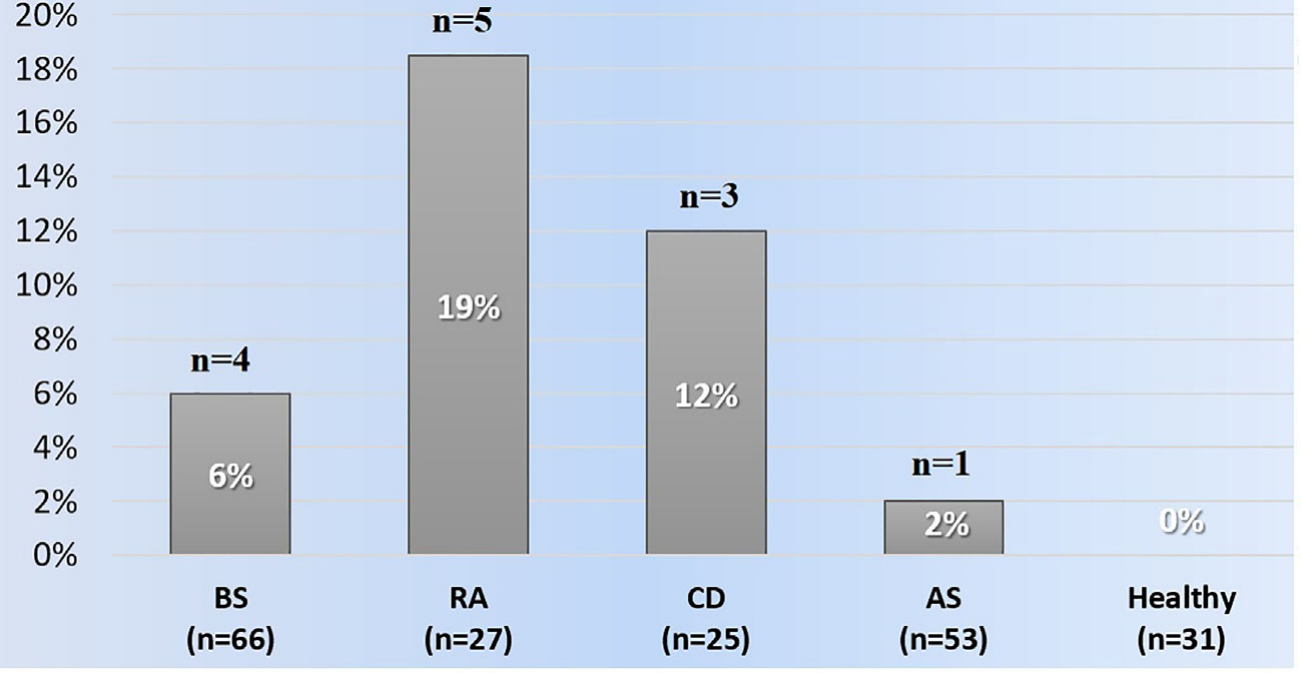
The frequencies of anti-infliximab antibodies among the groups. AS, Ankylosing spondylitis; BS, Behçet syndrome; CD, Crohn's disease; RA, Rheumatoid arthritis.

Anti-IFX antibodies were detected in four (6%) patients with Behçet syndrome, five (18.5%) with rheumatoid arthritis, one (2%) with ankylosing spondylitis, and three (12%) with Crohn’s disease. None of the healthy subjects had anti-IFX antibodies (**Figure 1**). Among the patients with ADAs, the four Behçet syndrome patients had received 5, 9, 16, and 19 infusions, five rheumatoid arthritis patients had received 5, 17, 24, 25, and 27 infusions, one ankylosing spondylitis patient had received 25 infusions, and three Crohn’s disease patients had received 7, 13, and 19 infusions before the time of serum sampling. Overall, 6/13 (46%) patients with anti-IFX antibodies were not on concomitant immunosuppressives or DMARDs.

Three of six rheumatoid arthritis patients, three of five Crohn’s disease patients and one of nine Behçet syndrome patients who had experienced a previous infusion reaction had anti-IFX antibodies whereas none of the four ankylosing spondylitis patients who had an infusion reaction had anti-IFX antibodies.

The median serum IFX trough level was significantly lower in patients with anti-IFX antibodies compared to those without antibodies in the whole group [2.32 μg/ml (IQR: 0.6–3.6) vs. 3.35 μg/ml (IQR: 1.63–5.6); p = 0.019]. The serum IFX trough level was lower than the cut off value in 6/13 patients with anti-IFX antibodies and in 25/158 without anti-IFX antibodies (46% vs. 16%; p = 0.015).

In 27 Behçet syndrome patients (2 with anti-IFX antibodies), we were able to get serum in at least two consecutive infusions. In all patients, the presence/absence of anti-IFX antibodies was consistent in serum samples collected at different time points.

We obtained serum samples before the infusion and at week 2 in five Behçet syndrome patients. Serum IFX level was below 1 μg/ml (0.34, 0.24, 0.68, 0.7, and 0.96 μg/ml) before IFX and above 1 μg/ml (15.4, 10.4, 8.6, 13.2, and 7.1 μg/ml) at week 2 in all of these five patients.

### Comparison of Behçet Syndrome Patients With and Without Anti-IFX Antibodies

Among the 66 patients with Behçet syndrome, anti-IFX antibodies were detected in 4 (6%) patients. All the patients with antibodies were male whereas there were 15 females among the 62 patients without antibodies. Mean age (SD) of patients with and without antibodies was 34 ± 4 and 37 ± 9 years, respectively. One of the four patients had had a previous infusion reaction. Three of the patients were using a DMARD concomitantly. Eight (13%) patients without antibodies experienced an infusion reaction and 43 (69%) were using a DMARD. Mean (SD) number of IFX infusions was 12 ± 6.4 in patients with antibodies and was 19.5 ± 14.8 in patients without antibodies.

Patients with Behçet syndrome with and without antibodies had similar and very low BSAS scores on the day of serum collection (mean ± SD BSAS score 4.5 ± 6.6 and 3.3 ± 4.3, p = 0.072, respectively).

Among the patients with antibodies, serum IFX trough level was higher than the cut-off value in three patients (75%). In the remaining 62 patients without anti-IFX antibodies, 53 patients (85.5%) had a serum IFX trough level higher than 1 μg/ml.

### Clinical Response to IFX During Follow-Up of Behçet Syndrome Patients

During a mean follow-up of 3 ± 0.5 years after serum sampling, 2/4 Behçet syndrome patients with anti-IFX antibodies had flares and 2 experienced infusion reactions. In the first patient, serum had been collected before the 16^th^ infusion. After two infusions of IFX, an infusion reaction occurred. However, he continued to receive IFX with premedication. At the 20^th^ infusion, he experienced an ocular relapse and was switched to adalimumab. In the second patient, serum had been collected before the 9^th^ infusion. He was switched to adalimumab due to active mucocutaneous lesions and joint involvement after four additional infusions. Both patients were still using adalimumab, for 3.5 and 2.5 years, in October 2019, when this study ended. The third patient with uveitis experienced an infusion reaction, however he was still continuing to use IFX for 4.5 years without a relapse. The last patient with a venous ulcer was off treatment and was in sustained remission for 2.5 years.

None of the 62 patients without anti-IFX antibodies discontinued therapy due to inefficacy. 45 patients were still on IFX. IFX was stopped due to remission in 16 patients and because of an infusion reaction in 1 patient. Overall, six infusion reactions occurred during follow-up (four without anti-IFX antibodies and two with anti-IFX antibodies; [4/62 (6.45%) vs. 2/4 (50%)].

## Discussion

Anti-IFX antibodies are associated with loss of treatment response and adverse events such as infusion reactions and paradoxical phenomenon such as induction of psoriasis, sarcoidosis and uveitis (21, 22). Testing for anti-IFX antibodies is being increasingly used to guide treatment strategies in patients with rheumatoid arthritis and Crohn’s disease (6, 9). IFX has become one of the main treatment options of Behçet syndrome (23, 24). Its indications have expanded to all manifestations of Behçet syndrome and are not limited to refractory patients (12).

As far as we know there was only one previous study on the immunogenicity of IFX inhibitors in Behçet syndrome, and different from that study, we have investigated the frequency of anti-IFX antibodies in patients with Behçet syndrome receiving IFX, together with diseased controls and healthy subjects to better assess the magnitude of the problem of immunogenicity. Our study showed that the frequency of anti-IFX antibodies in patients with Behçet syndrome were lower than in patients with rheumatoid arthritis and Crohn’s disease receiving IFX and was comparable to that observed in patients with ankylosing spondylitis. Among the four Behçet syndrome patients with anti-IFX antibodies, one had to discontinue IFX due to inefficacy, one experienced a relapse, and one patient experienced both. On the other hand, none of the 62 Behçet syndrome patients without anti-IFX antibodies had to discontinue IFX due to inefficacy and 4 of them had infusion reactions. Since majority of the patients with Behçet syndrome had major organ involvement, a comparison of immunogenicity between patients with major organ involvement and only mucocutaneous involvement was not possible. Interestingly all Behçet syndrome patients with anti-IFX antibodies were men. However, we are not aware of any data on the impact of gender on IFX immunogenicity. This may simply be associated with the high proportion of men (77%) receiving IFX, since Behçet syndrome runs a more severe disease course in men.

The prevalence of anti-IFX antibodies varies widely across inflammatory diseases and different types of TNFi (5). In addition, the duration of therapy, continuous vs. intermittent use, concomitant use of DMARDs, high vs. low biologic doses are factors associated with immunogenicity (6, 25). These factors may explain the variation across studies in the frequency of anti-IFX antibodies. A systematic literature review showed that the frequency of anti-IFX antibodies ranges from 8% to 62% in rheumatoid arthritis patients, 6.1% to 69% in ankylosing spondylitis patients and 3% to 83% in Crohn’s disease patients (5). In our study, the proportions of patients with anti-IFX antibodies were 18.5% in rheumatoid arthritis, 2% in ankylosing spondylitis and 12% in Crohn’s disease. These somewhat low frequencies may be associated with our cross-sectional study design. Mean number of IFX infusions before serum sampling was 19 ± 14 SD in Behçet syndrome patients, 21 ± 13 in rheumatoid arthritis patients, 33 ± 18 in ankylosing spondylitis patients, and 19 ± 21 in Crohn’s disease patients. Since this was not a prospective longitudinal study, it is possible that some of the patients who developed ADAs may have discontinued IFX due to loss of efficacy or infusion reactions, causing us to detect a lower frequency of ADAs in this study.

Underlying disease itself can be a factor in the context of immunogenicity (6). Patients with autoimmune diseases may have an immune system prone to altered immunological tolerance to self-proteins and this may render them more prone to develop ADAs (26). This may be an explanation for the lower frequency of ADAs in our patients with Behçet syndrome and ankylosing spondylitis, since both are not predominantly autoimmune conditions.

Differences in concomitant drug use may be another and perhaps more important factor underlying the difference in immunogenicity between diseases. A meta-analysis of studies reporting on the immunogenicity of TNFi therapy in immune-mediated inflammatory diseases showed 41% lower frequency of detectible ADAs among patients that used concomitant DMARDs compared with those that did not use these agents (RR: 0.59, 95% CI 0.50 to 0.70) (27). In our study, 70% of Behçet syndrome patients, 63% of rheumatoid arthritis patients, 88% of Crohn’s disease patients, and 13% of ankylosing spondylitis patients were using an immunosuppressive or a DMARD at the time of serum sampling. Even though less ankylosing spondylitis patients were using concomitant DMARDs, they had a low frequency of ADAs compared to other groups. On the contrary our Crohn’s disease patients had a high frequency of ADAs despite a higher rate of concomitant immunosuppressive use. These observations suggest that concomitant immunosuppressive use may not be an important factor in the low frequency of anti-IFX antibodies that we observed in our Behçet syndrome patients.

The currently used assays for determining ADAs and serum drug levels seem to be reliable (28–30). A study from Spain that included patients with inflammatory bowel diseases compared four ELISA-based assays for serum IFX trough levels, and three ELISA-based assays and one RIA-based assay for detection of anti-IFX antibodies (31). The authors found “almost perfect” concordance. Similarly, we observed good concordance between the results obtained in consecutive measurements in a subgroup of Behçet syndrome patients.

In conclusion, our study showed that immunogenicity to IFX is not frequent in Behçet syndrome, but may be associated with adverse events and relapses, when present. Our main limitation was the relatively small number of patients. More patients, longer observation time and longitudinal assessment are needed to assess the association of these findings with clinical response and adverse events.

## Data Availability Statement

The original contributions presented in the study are included in the article/supplementary material. Further inquiries can be directed to the corresponding author.

## Ethics Statement

The studies involving human participants were reviewed and approved by the ethics committee of Cerrahpasa Medical School, Istanbul University-Cerrahpasa (83045809/852). The patients/participants provided their written informed consent to participate in this study.

## Author Contributions

SE: conceptualization, methodology, formal analysis, and writing—original draft. FA-M: investigation and visualization. YO: investigation and visualization. FO: investigation and visualization. ON: investigation and visualization. DC: investigation and visualization. VH: visualization and writing—review and editing. IH: investigation and visualization. AC: investigation and visualization. HY: conceptualization, methodology, and writing—review and editing. GH: conceptualization, methodology, formal analysis, writing–original draft, writing—review and editing, and supervision. All authors contributed to the article and approved the submitted version.

## Funding

The study was supported by Istanbul University, Scientific Research Projects Coordination Unit (Project no: 57420).

## Conflict of Interest

GH has received grant/research support from Celgene and has served as a speaker for AbbVie, Celgene, Novartis, and UCB Pharma.

The remaining authors declare that the research was conducted in the absence of any commercial or financial relationships that could be construed as a potential conflict of interest.
